# Metabolic expenditure of submaximal locomotion in naked mole-rats (*Heterocephalus glaber*) and Damaraland mole-rats (*Fukomys damarensis*)

**DOI:** 10.1242/jeb.249875

**Published:** 2025-06-25

**Authors:** Jack E. Thirkell, Nigel C. Bennett, Daniel W. Hart, Chris G. Faulkes, Monica A. Daley, Steven J. Portugal

**Affiliations:** ^1^ Department of Biological Sciences, School of Life and Environmental Sciences, Royal Holloway University of London, Egham, Surrey TW20 0EX, UK; ^2^ Mammal Research Institute, University of Pretoria, Hatfield 0028, South Africa; ^3^ Department of Zoology and Entomology, University of Pretoria, Hatfield 0028, South Africa; ^4^ School of Biological and Behavioural Sciences, Queen Mary University of London, London E1 4NS, UK; ^5^ Department of Ecology and Evolutionary Biology, University of California, Irvine, Irvine, CA 92697, USA; ^6^ Department of Biology, University of Oxford, Oxford OX1 3SZ, UK

**Keywords:** African mole-rats, Bathyergidae, Metabolic rate, Movement physiology, Respirometry, Rodent treadmill, Subterranean

## Abstract

Despite extensive studies on the physiology of subterranean rodents, there is comparatively little work documenting the energetics specifically associated with their locomotory energetics. The energetic cost associated with burrowing is great and, in part, explains why subterranean species often maintain their burrows and tunnels across generations. Indeed, the digging metabolic rate of five African mole-rats has been determined to be between three and five times higher than their respective resting metabolic rate, yet the energetic cost of non-digging locomotion (i.e. walking) has not been recorded. Digging in most subterranean species tends to lead to specialisation of the forelimbs and teeth, which may significantly affect the energetic cost associated with non-digging locomotion. Unlike many comparably sized burrowing and tunnelling mammals, African mole-rats appear, superficially at least, to have almost identical forelimbs and hindlimbs. This study explored the locomotory energetics associated with sustained submaximal locomotion (i.e. fast walking; 10 cm s^−1^) in two African mole-rat species (*Fukomys damarensis* and *Heterocephalus glaber*), utilising open-flow respirometry and a small animal treadmill. The mean locomotory energetic rate for *F. damarensis* was a near 1-fold increase (91.4%) above resting metabolic rate and a 2-fold increase (203.2%) for *H. glaber*. Net cost of transport was higher overall for *H. glaber* (2.9±0.6 ml O_2_ kg^−1^ m^−1^) than for *F. damarensis* (2.4±0.5 ml O_2_ kg^−1^ m^−1^). A trade-off likely exists between limb specialisation for digging and economic locomotion, and thus for most obligately subterranean species, locomotion represents an energetic investment.

## Teaser

**Summary:** Examination of walking energetics in African mole-rats reveals higher metabolic costs in *Heterocephalus glaber* than in *Fukomys damarensis*, suggesting a trade-off between digging specialisation and locomotion efficiency.

## INTRODUCTION

At least 250 extant rodent species have evolved to occupy a subterranean environment, spending most of their lives in self-constructed burrow systems (Begall et al., 2007). African mole-rats (Bathyergidae) are one such group of small (30–1800 g) rodents found in sub-Saharan Africa. While a subterranean lifestyle offers enhanced protection during periods of rest, hibernation, breeding and nursing, it does not come without costs; intermittent resources, increased energetic costs associated with digging, high humidity, reduced gas ventilation and low atmospheric oxygen are just some of the specific challenges that these underground environments present (Nevo, 1995; Burda et al., 2007; Jonz et al., 2016).

Subterranean systems – burrows and tunnels – have historically been considered hypoxic environments. Assessment of the air within the burrows of naked mole-rats (*Heterocephalus glaber*) showed that oxygen concentrations could reach as low as 8% and carbon dioxide as high as 10% in burrows, driven predominantly by the cohabitation of a large numbers of individuals within a relatively confined space, little air movement and impeded gas exchange through the substrate (Bennett and Faulkes, 2000). Yet, research looking at the environmental conditions within burrows of *Georychus capensis* and *Fukomys damarensis* also identified that oxygen and carbon dioxide concentrations may not be greatly different to surface values (Roper et al., 2001), although measurement of burrow gas composition needs further study, especially within nest chambers (Buffenstein et al., 2021). This would indicate that while African mole-rats appear to be highly tolerant of fluctuating gas concentrations, they do not necessarily live in a chronic state of hypoxia (low oxygen) or hypercapnia (high carbon dioxide).

Low oxygen and high carbon dioxide concentrations may act synergistically with irregularly distributed food resources and elevated costs of digging and burrow maintenance to increase metabolic expenditure for subterranean mammals (Faulkes and Bennett, 2013). Some species, including *H. glaber*, appear to acclimate to these conditions by significantly reducing metabolic rate (39.6±2.9 to 12±0.3 ml O_2_ min^−1^ kg^−1^) and the rate of ventilation (1.412±244 to 417±62 ml min^−1^ kg^−1^) in response to artificially reduced oxygen concentrations of 7% (Pamenter et al., 2015; see also Merchant et al., 2024a). While African mole-rats, most notably *H. glaber*, have been subject to extensive study for their many interesting geno-phenotypic characteristics (Jarvis, 1981; Park et al., 2008; Seluanov et al., 2009; Liang et al., 2010; Edrey et al., 2011), other aspects of their physiology, for example, their locomotory attributes, have been poorly studied.

Subterranean species are arguably faced with some of the greatest levels of physical resistance to movement. Even so, different behaviours are exhibited depending on the substrate and its density. Species such as African mole-rats and ground squirrels (*Callospermophilus lateralis*) tunnel their way through the compacted ground, actively redistributing the surrounding substrate, in contrast to species such as Namib Desert golden moles (*Eremitalpa granti namibensis*), which use a swimming motion to move rapidly through the loose sandy substrate (Seymour et al., 1998). Furthermore, Gans (1975) hypothesized that enhanced subsurface locomotory performance and decreased metabolic expenditure are a product of body elongation. This provides the basis of correlations, identified by Lee (1998), of the adaptive morphology of burrowing reptiles. The energetic cost associated with burrowing is great; the digging metabolic rate (DMR) of five African mole-rat species (*H. glaber*, *F. damarensis*, *Fukomys mechowii*, *Heliophobius argenteocinereus* and *G. capensis*) has been determined to be between three and five times greater than their respective resting metabolic rate (RMR) (Du Toit et al., 1985; Lovegrove, 1989; Zelová et al., 2007). Similarly, two species of pocket gophers (*Thomomys talpoides* and *Thomomys bottae*) have DMR to RMR ratios of 2.47 and 4.88, respectively, which may explain in part why tunnel systems of subterranean rodents are used and maintained, often across generations (Bennett and Faulkes, 2000; Cameron, 2000). While the energetic costs of digging in African mole-rats have been determined to be typically greater than those of other subterranean rodents (Bennett and Faulkes, 2000), the energetics of general African mole-rat non-digging (i.e. fast walking) locomotion have not been studied. Holtze et al. (2018) noted that the subterranean environment that mole-rats occupy has been studied relatively little, in particular the energetic costs associated with locomotion.

All species of African mole-rats, with the exception of *Bathyergus suillus* and *Bathyergus janetta*, are tooth-diggers as opposed to scratch-diggers, which likely acts as a strong selection pressure for enlarged dentition and powerful jaw musculature (McIntosh and Cox, 2016; Merchant et al., 2024b). Whereas similarly sized mammals would generally use their forelimbs or hindlimbs to excavate substrate as they expand tunnel systems, African mole-rats use their limbs and feet to sweep excavated material out of tunnels. Both the forelimbs and hindlimbs of tooth-diggers appear to be short, sitting directly below the body, and exhibit no appreciable morphological specialisation for digging. The lack of adaptation to digging in their limbs perhaps explains why mole-rats appear adept at general agile movement. Conversely, digging in other subterranean species, for example, European moles (*Talpa europaea*) and golden moles (Bronner and Bennett, 2005), tends to lead to specialisation of the forelimbs (Polly, 2007). Unlike many comparably sized burrowing and tunnelling mammals, tooth-digging African mole-rats appear, superficially at least, to have almost identical forelimbs and hindlimbs.

African mole-rats, like many subterranean species to a certain extent, have been freed of the many constraints normally placed on most animals when they move; a low centre of gravity and support from tunnel walls affords them great support and stability. An ultimate reason why they might have unusual movement physiology is that they have few concerns over pitch or roll stability because they move in such enclosed spaces. Nonetheless, they must support their body weight against gravity to maintain posture during locomotion, like all other mammals. Generating sufficient vertical force to support body weight against gravity is the main determinant of ground reaction force magnitudes in locomotion, and the cost of activating muscles to generate force is the primary determinant of the energetic cost of locomotion in terrestrial mammals (Taylor and Heglund, 1982; Heglund and Taylor, 1988; Kram and Taylor, 1990). While vertical force demands remain, stability demands are likely to be lower in subterranean species; consequently, it remains unclear whether locomotor energetics differ between subterranean and overground locomotion. Additionally, there is sparse published literature explaining how the physiology of subterranean species responds to intermittent hypoxia, and how this facilitates a largely unencumbered subterranean existence. Knowledge of subterranean locomotor energetics could, therefore, potentially inform our understanding of movement in different environments, with applications in artificial intelligence and robotics.

Our study aimed to determine the locomotory energetics associated with sustained submaximal (i.e. not pushed to their energetic limit) locomotion (10 cm s^−1^) in two highly social African mole-rat species (*F. damarensis and H. glaber*), utilising open-flow respirometry and a commercially available small animal treadmill. Measuring the energetics of locomotion represents the first insight into the locomotory energetics of this mammalian clade. Specifically, this study explored whether these species exhibit an increase in metabolic rate over time in response to sustained locomotory activity, and determined the increase in metabolic rate during exercise compared with RMR. Lastly, we assessed whether the allometric scaling of locomotory energetics differs in response to sustained locomotion; does body mass determine the metabolic response to sustained locomotion? *Fukomys damarensis* and *H. glaber* are unusual in that not only are they the only two eusocial mammals but as such they also exhibit an allometric division of labour, whereby colony roles tend to be determined by the body mass of an individual (Bennett and Jarvis, 1988; Scantlebury et al., 2006; Faulkes and Bennett, 2013; but see also Gilbert et al., 2020). Thus, animals with smaller body mass generally engage in colony maintenance type roles (e.g. care of young, digging and foraging), while animals with larger body mass engage in reproductive and defence type roles. It might be expected that differences in the metabolic demands of these role-associated behaviours will elicit different metabolic responses to sustained locomotion. This difference in metabolic responses to locomotion may reflect an increase in allometric scaling of locomotory energetics, in both species, with increasing time spent moving.

## MATERIALS AND METHODS

### Study animals

The locomotory energetics of 13 *Fukomys damarensis* (Ogilby 1838) and 10 *Heterocephalus glaber* Rüppell 1842 were measured while moving at a submaximal speed (10 cm s^−1^) on a 0 deg inclination (Table S1). The speed was selected at which a stable, steady gait could be consistently maintained. At higher speeds, the animals began to struggle – their gait became awkward and they often drifted toward the back of the chamber. Both species of mole-rat could physically move faster but not sustain a stable gait over time at these higher speeds without tripping or stumbling. Thus, the chosen speed represents the upper limit at which a consistent, long-duration gait was achievable across all individuals of both species. Captive populations of these species were maintained in their respective colonies in the Department of Zoology and Entomology at the University of Pretoria (UoP). Throughout this study, animals were provided with appropriate nesting material and fed *ad libitum* on sweet potatoes, which were replaced daily. The animals were maintained in large polyurethane containers, housed in a climate-controlled laboratory that maintained an ambient temperature (*T*_a_) between 23 and 25°C, a relative humidity of 40–60% and a light cycle set to 12 h light:12 h dark (Ivy et al., 2020; Jacobs et al., 2023). Animals were fasted for >12 h prior to assessments, to ensure a postabsorptive state and exclude the potential influence of digestion on metabolic activity (Šumbera, 2019; Wallace et al., 2021). Only adult animals that were considered to be neither pregnant nor lactating were assessed (N.C.B., personal observation). Despite an apparent absence of circadian rhythms among these species (Bennett and Faulkes, 2000), for continuity with other metabolic studies on African mole-rats and to follow established protocols, we conducted all assessments between 08:00 h and 18:00 h, to mitigate against the potential effects of endogenous metabolic rhythms. Experimental procedures involving live animals and data collection described herein were approved by Royal Holloway University of London and the UoP Animal Ethics Committee (Ref. EC004-19). The study was conducted in accordance with appropriate institutional and national guidelines.

### Experimental procedure

Locomotory energetics were determined through the measurement of the rate of oxygen consumption (*V̇*_O_2__) and carbon dioxide production (*V̇*_CO_2__), using an open-flow respirometer (Sable Systems International, Las Vegas, NV, USA) and a small animal treadmill (Panlab/Harvard Apparatus LE8700). The treadmill was coupled with a control unit (Panlab/Harvard Apparatus), which controlled the speed on the treadmill belt and could measure additional parameters (e.g. distance travelled, shock time and shock intensity – although such functions were not utilised in this study). The ambient room temperature was 30±2°C for *H. glaber* and 25±4°C for *F. damarensis*. The gait of both species was confirmed as a walk, through video analysis of the footfall pattern and duty factor (Alexander, 1977). Mass-specific cost of transport (COT; ml O_2_ kg^−1^ m^−1^) was calculated as the rate of oxygen consumption (in ml O_2_ kg^−1^ min^−1^) over treadmill speed (m min^−1^), yielding the oxygen consumed per unit of mass per distance travelled (ml O_2_ kg^−1^ m^−1^) (e.g. Schaeffer et al., 2005).

Each respirometry assessment lasted approximately 65 min and consisted of a 10 min baseline to assess ambient O_2_ level, a 26 min metabolic assessment, followed by a further 10 min baseline to reassess ambient O_2_. The respirometer consisted of a 4.5 l acrylic container, fitted with 4 mm inlet and outlet ports. The respirometry chamber was positioned on a wooden frame that was fastened along the long edges of the treadmill, and across the width of the treadmill with the addition of dense brushes to reduce air leakage. The outside air was pulled through the respirometer at a flow rate of 1400 ml min^−1^, resulting in an approximate flush-out rate of 3 min 20 s. The analog outputs of O_2_ (%), CO_2_ (%), flow rate (ml min^−1^), relative humidity (%), barometric pressure (kPa) and temperature (°C) were recorded concurrently using a universal interface (UI2, Sable Systems International). These measurements were sampled (1 Hz) and monitored in real-time using ExpeData software (Sable Systems International), which enabled the progress and stability of each animal's respirometry trace to be visually assessed. Additionally, this enabled the manual addition of markers on the trace to note times of aberrant behavioural observations or external confounding factors. Through real-time monitoring of the gas traces, we were able to safeguard against potentially dangerous spikes in CO_2_ or drops in O_2_, at which point the assessment would have been terminated. Body mass (g) was measured immediately preceding each assessment using Oertling electronic weigh scales (Oertling, Birmingham, UK).

Incurrent airflow was controlled using a flow regulating pump (SS-4, Sable Systems International), calibrated against a certified mass flow meter (FoxBox, Sable Systems International), placed downstream of the respirometry chamber. Fractional concentration of O_2_ was measured using an oxygen analyser (FC-10a, Sable Systems International), which was calibrated to ambient air O_2_ concentration (20.95%) before each trial. Fractional concentration of CO_2_ was measured using a carbon dioxide analyser (CA-10a, Sable Systems International), and relative humidity measured using a water vapour analyser (RH-300, Sable Systems International). Barometric pressure and temperature were measured from inbuilt sensors in the FC-10a oxygen analyser. Anhydrous Indicating Drierite™ was used to scrub atmospheric water from the excurrent air between the water vapour and carbon dioxide analysers, and again between the CO_2_ scrubber and the oxygen analyser (W. A. Hammond Drierite Company Ltd). Drierite was fully saturated and recharged prior to the first use, following the recommendation of White et al. (2006a). CO_2_ was scrubbed from the excurrent air between the CO_2_ and O_2_ analysers (Soda Lime, Sigma-Aldrich, Merck KGaA, Darmstadt, Germany).

**Fig. 1.**
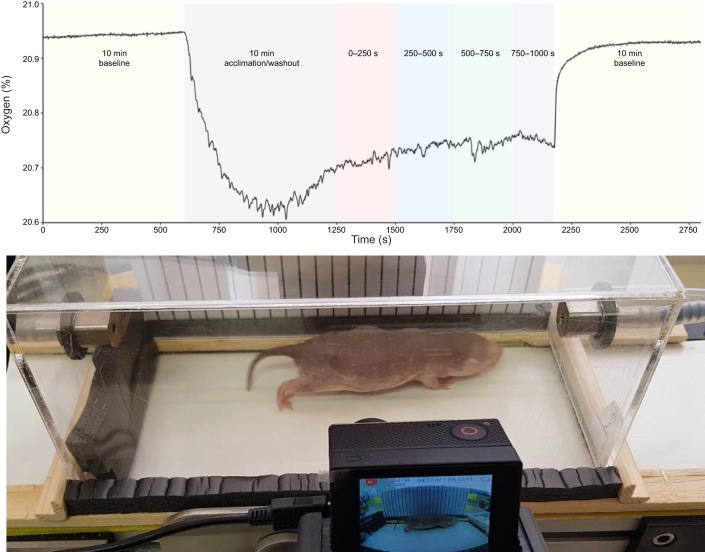
**Experimental set-up.** Top: a representative respirometry trace depicting the locomotory energetics associated with sustained submaximal locomotion in a species of African mole-rat (*Heterocephalus glaber*). The trace is segmented by two 10 min baseline periods, a 10 min acclimation/washout period and four 250 s analysis regions. Bottom: a photograph of *H. glaber* on the small animal treadmill during respirometry assessment of locomotory energetics.

Data, once exported from ExpeData, were processed in Matlab (version 9.6, The MathWorks Inc., Natick, MA, USA). O_2_ and CO_2_ were corrected for baseline drift and any time lag between these two variables (due to the delay in airflow between analysers) was corrected using cross-correlation. The fractional O_2_ signal was corrected for the removal of CO_2_ (O_2__corrected), the fractional CO_2_ signal was corrected for the removal of water vapour (CO_2__corrected), and the flow rate was corrected to Standard Temperature and Pressure (STP) conditions. The first 10 min the animal was on the treadmill was considered a sufficient acclimation period (Fig. 1), which also enabled air in the respirometer and tubing to be fully recycled (i.e. flush out rate), and thus was omitted from analyses. The remaining ∼16 min that the animals were on the treadmill were split into four 250 s periods (Fig. 1). The time periods were chosen based on a combination of washout and equilibrium times. For analysis, we picked windows where the O_2_ trace had clearly stabilised following (speed) transitions. They were not entirely arbitrary, aside from the minimum time – the same criteria were applied across animals, based on visual inspection of when the respirometry signal had plateaued. Mean *V̇*_O_2__ and *V̇*_CO_2__ were calculated using the formulae:
(1)

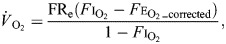

(2)

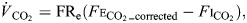
where *F*i and *F*e are incurrent and excurrent fractional concentrations (%) of O_2_ and CO_2_ (Lighton, 2008) and FR is flow rate. The ratio of *V̇*_CO_2__ to *V̇*_O_2__ determined the respiratory quotient (RQ) (Lighton, 2008). Unless otherwise stated, *V̇*_O_2__ is expressed as the locomotory metabolic rate (ml O_2_ h^−1^) and is presented as the mean±s.d., corrected to STP conditions.

### Statistical analyses

Separate repeated measure analyses of variance (ANOVA) determined whether, for both *F. damarensis* and *H. glaber*, there were significant differences in the mean locomotory energetic rate across the four time periods. Furthermore, to identify where differences arise, estimated marginal means (EMMs) using a Tukey adjustment enabled pairwise comparisons between each time period. Body mass was also incorporated into statistical modelling as a covariate, with simple one-way repeated analyses of covariance (ANCOVA) determining whether there was an interaction between body mass and locomotory energetics and whether this varied with time. Similarly, estimated marginal trends (EMTs) with a Tukey adjustment enabled pairwise comparisons between each time period (i.e. differences in the allometric scaling of locomotory energetics between time periods). Differences in locomotory metabolic rate, body mass and COT between the species and between sexes were compared using *t*-tests and ANOVA. Data are presented as means±s.e.m unless otherwise stated. Bonferroni corrections to alpha were applied where appropriate to account for multiple tests. All calculations and statistical analyses were performed in R statistical software (v.3.5.21). EMMs and EMTs were calculated using the R package *emmeans* (v.1.8.3; https://CRAN.R-project.org/package=emmeans).

## RESULTS

Combining all data per species, *F. damarensis* had a significantly higher locomotory metabolic rate than *H. glaber* (*F. damarensis* 4.56±0.63 ml O_2_ h^−1^, *H. glaber* 2.07±0.33 ml O_2_ h^−1^; *t*-test, *t*_90_=−13.28, *P*<0.0001). Overall, male *F. damarensis* had a higher locomotory metabolic rate than females, when data from all time points were combined (males, *N*=28, 293.9±27.3 ml O_2_ h^−1^; females, *N*=24, 249.9±16.5 ml O_2_ h^−1^; *t*-test, *t*_50_=−2.6, *P* <0.05), likely due to their significantly higher body mass (males 129.8±5.2 g, females 108.3±3.7 g; *t*-test, *t*_50_=−6.5, *P*<0.01). This difference in locomotory metabolic rate between male and female *F. damarensis* did not persist when mass-corrected values of locomotory metabolic rate were compared (males 0.04±0.01 ml O_2_ h^−1^ g^−1^, females 0.04±0.01 ml O_2_ h^−1^ g^−1^; *t*-test, *t*_50_=0.24, *P*=0.8). In *H. glaber*, there was no significant difference in locomotory metabolic rate between the sexes when all time points were combined (males, *N*=20, 117.3±7.42 ml O_2_ h^−1^, females, *N*=20, 131.8±7.75 ml O_2_ h^−1^; *t*-test, *t*_38_=1.35, *P*=0.18), likely due to the lack of significant difference in body mass between the sexes (males 41.2±2.02 g, females 43.6± 1.30 g; *t*-test, *t*_38_=−0.99, *P*=0.32). Mass-corrected values of locomotory metabolic rate for *H. glaber* were 0.04±0.01 ml O_2_ h^−1^ g^−1^ for males and 0.05±0.01 ml O_2_ h^−1^ g^−1^ for females. Mass-corrected locomotory metabolic rate did show a significant difference between the sexes in *H. glaber*, with females having a higher locomotory metabolic rate (*t*-test, *t*_38_=2.67, *P*<0.05).

Combining all data per species, *F. damarensis* had a significantly lower COT than *H. glaber* (*t*-test, *t*_90_=−5.62, *P*<0.0001; *F. damarensis* 6.37±0.88 O_2_ kg^−1^ m^−1^, *H. glaber* 8.01±1.14 O_2_ kg^−1^ m^−1^). In both species for all data combined, COT did not differ significantly over the time periods (*F. damarensis*, ANOVA, *F*_51_=0.64, *P*=0.94; *H*. *glaber*, ANOVA, *F*_39_=0.6, *P*=0.95). While there was no overall difference in COT across all time periods between the sexes in *F. damarensis* (males 6.33±1.22 O_2_ kg^−1^ m^−1^, females 6.42±1.31 ml O_2_ kg^−1^ m^−1^; *t*-test, *t*_50_=0.24, *P*=0.81), COT in female *H*. *glaber* was significantly higher than that in males (males 7.48±1.67 O_2_ kg^−1^ m^−1^, females 8.86±1.98 ml O_2_ kg^−1^ m^−1^; *t*-test, *t*_38_=2.68, *P*<0.01). Full *V̇*_O_2__, *V̇*_CO_2__, RQ and COT data can found be in Table 1.

**Table 1.** *V̇*_O_2__, *V̇*_CO_2__, RQ and mass-specific COT in two African mole-rat species, *Fukomys damarensis and Heterocephalus glaber*

Species	Time segment (s)	*V̇*_O_2__ (ml O_2_ min^−1^)	*V̇*_CO_2__ (ml CO_2_ min^−1^)	RQ	COT (ml O_2_ kg^−1^ m^−1^)
*F. damarensis*	0–250	4.86±0.29	3.40±0.21	0.70±0.01	6.8±1.88
	250–500	4.58±0.27	3.22±0.22	0.70±0.01	6.4±1.85
	500–750	4.50±0.30	3.12±0.22	0.69±0.01	6.3±1.81
	750–1000	4.31±0.34	2.96±0.25	0.68±0.01	6.1±1.72
*H. glaber*	0–250	2.27±0.36	1.70±0.15	0.75±0.58	8.7±2.52
	250–500	2.13±0.34	1.61±0.14	0.75±0.58	8.1±2.35
	500–750	1.98±0.31	1.50±0.13	0.75±0.57	7.9±2.27
	750–1000	1.92±0.30	1.42±0.12	0.74±0.57	7.4±2.34

Rate of oxygen consumption (*V̇*_O_2__) and carbon dioxide production (*V̇*_CO_2__), respiratory quotient (RQ) and mass-specific cost of transport (COT) data (means±s.e.m.) are presented for each time segment for *F. damarensis* (*N*=13) and *H. glaber* (*N*=10).

### Does sustained submaximal locomotion increase metabolic rate over time?

**Fig. 2.**
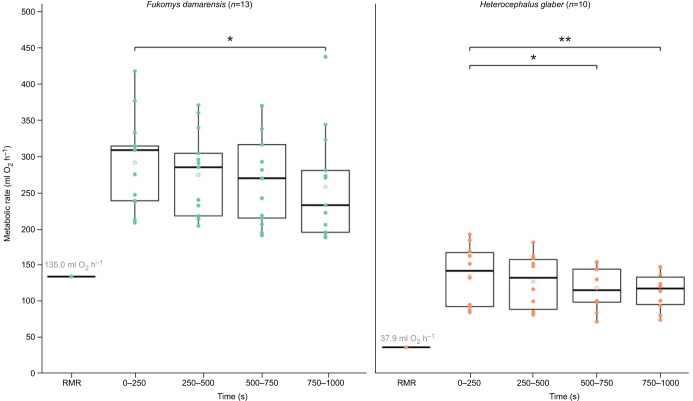
**The locomotory energetics of sustained submaximal locomotion (10 cm s^−1^) in two species of African mole-rats, *Fukomys damarensis* and *H. glaber*, at four time periods.** Data are shown as the distribution of locomotory energetics values, including the lower quartile (Q1) and upper quartile (Q3) values, in addition to the mean (open circle) and median (bold horizontal black line). Included also, for comparison, is the mean resting metabolic rate (RMR; ml O_2_ h^−1^) of both species, calculated and weighted by the sample size of the respective published studies [*F. damarensis*, *N*=6 (Lovegrove, 1986; Bennett et al., 1992; Scantlebury et al., 2006; Ivy et al., 2020); *H. glaber*, *N*=3 (McNab, 1966; Buffenstein and Yahav, 1991)]. Asterisks denote significance (*P<0.05, **P<0.01).

One-way repeated measures ANOVA identified that there were significant differences in the mean locomotory energetics between the four time periods, for both *F. damarensis* (*F*_3,36_=3.56, *P<*0.05; Table 2 and Fig. 2) and *H. glaber* (*F*_3,27_=5.16, *P<*0.01; Table 3 and Fig. 2). Specifically, *post hoc* analyses – EMTs with a Tukey adjustment – revealed that for *F. damarensis*, there was a significant difference in the mean locomotory energetic rate between the first (0–250 s) and fourth (750–1000 s) time period (EMM; *P*<0.05; Table 2 and Fig. 2). Similarly, there were significant differences between the first and fourth time period for *H. glaber* (EMM; *P*<0.01; Table 3 and Fig. 2), in addition to the first (0–250 s) and third (500–750 s) time period (EMM; *P*<0.05; Table 3 and Fig. 2). Despite a trend towards decreasing mean locomotory energetic rate over time, no further significant differences in mean metabolic rate were observed (*P*>0.05; Fig. 2, Tables 2 and 3).

**Table 2.** One-way analysis of variance indicates that time is a significant determinant of locomotory energetics in *F. damarensis*

	Estimate	s.e.	d.f.	SS	MS	*t-*ratio	*P*	*F*	Pr(>*F*)
Time			3	7387	2462.3			3.561	0.023*
Residuals			36	24,896	691.5				
Contrast between time periods						* *			
1st–2nd	16.84	10.3	36			1.633	0.374		
1st–3rd	21.75	10.3	36			2.109	0.170		
1st–4th	33.13	10.3	36			3.212	0.014*		
2nd–3rd	4.91	10.3	36			0.476	0.964		
2nd–4th	16.29	10.3	36			1.579	0.403		
3rd–4th	11.37	10.3	36			1.103	0.690		

Specifically, estimated marginal means with a Tukey adjustment identified that the only pairwise comparison with significantly different mean locomotory energetics was between the first and fourth time period. *P*-value adjustment, Tukey method for comparing a family of four estimates. d.f., degrees of freedom; SS, sum of squares; MS, mean square. Asterisks indicate significance.

**Table 3.** One-way analysis of variance indicates that time is a significant determinant of locomotory energetics in *H. glaber*

	Estimate	s.e.	d.f.	SS	MS	*t-*ratio	*P*	*F*	Pr(>*F*)
Time			3	2654	884.8			5.159	0.006*
Residuals			27	4631	171.5				
Contrast between time periods						* *	* *	* *	* *
1st–2nd	8	5.86	27			1.367	0.530		
1st–3rd	16.9	5.86	27			2.885	0.036*		
1st–4th	21.08	5.86	27			3.6	0.007*		
2nd–3rd	8.89	5.86	27			1.518	0.441		
2nd–4th	13.08	5.86	27			2.233	0.140		
3rd–4th	4.19	5.86	27			0.715	0.890		

Specifically, estimated marginal means with a Tukey adjustment identified that the only pair wise comparison with significantly different mean locomotory energetics were between the first and fourth time period. *P*-value adjustment, Tukey method for comparing a family of four estimates. Asterisks indicate significance.

### What are the locomotory energetics associated with submaximal locomotion over and above RMR?

The mean RMR of *F. damarensis* and *H. glaber* was calculated to be 135.0 ml O_2_ h^−1^ and 37.9 ml O_2_ h^−1^, respectively (Fig. 2). The values were calculated and weighted by the sample size of the respective published studies [*F. damarensis*; *N*=6 (Lovegrove, 1986; Bennett et al., 1992; Scantlebury et al., 2006; Ivy et al., 2020); *H. glaber*, *N*=3 (McNab, 1966; Buffenstein and Yahav, 1991)]. As there was no significant difference in the respective locomotory energetics of either species across the three final time periods, the lowest value was assumed to be an appropriate representation of the energetics associated with submaximal locomotion. Thus, the mean locomotory energetic rate for *F. damarensis* was 258.4±74.4 ml O_2_ h^−1^, representing a near 1-fold increase (91.4%) above RMR. Meanwhile, the mean locomotory metabolic rate for *H. glaber* was 114.9±26.0 ml O_2_ h^−1^, representing a 2-fold increase (203.2%) above RMR.

### Does allometric scaling differ in response to sustained locomotion, i.e. does body mass determine the metabolic response to sustained locomotion?

**Fig. 3.**
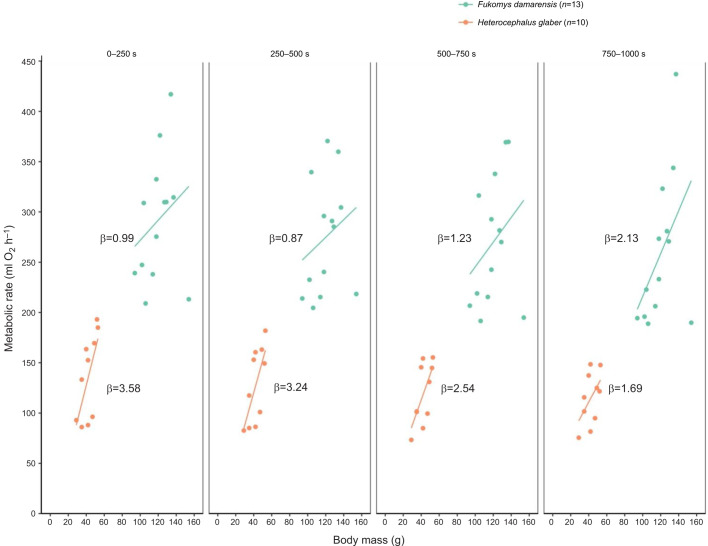
**Allometric scaling.** The allometric scaling of locomotory energetics (ml O_2_ h^−1^) associated with sustained submaximal locomotion (10 cm s^−1^) in two species of African mole-rats (*F. damarensis* and *H. glaber*), at four time periods. No significant differences in the scaling exponents (β) between each species' respective time periods were identified.

One-way ANOVA determined that while time was a significant determinant of locomotory energetics for *F. damarensis* (ANCOVA; *F=*3.74, *P<*0.05; Table 4 and Fig. 3) and both time (ANCOVA; *F=*6.16, *P<*0.01; Table 5 and Fig. 3) and body mass (ANCOVA; *F=*6.45, *P<*0.05; Table 5 and Fig. 3) were significant determinants for *H. glaber*, a significant interaction effect between these traits was not observed for either species (*P>*0.05; Tables 4 and 5). The lack of significant effects was confirmed by EMTs, which did not identify any significant difference in the allometric scaling of locomotory energetics between any of the four time periods (EMT; *P>*0.05; Tables 4 and 5).

**Table 4.** One-way analysis of covariance indicates that while time is a significant determinant of locomotory energetics in *F. damarensis*, body mass is not

	Estimate	s.e.	d.f.	SS	MS	*t-*ratio	*P*	*F*	Pr(>*F*)
Body mass			1	22,272	22,272			1.571	0.236
Time			3	7387	2462.3			3.742	0.020*
Time×Body mass			3	3178	1059.4			1.61	0.206
Contrast									
1st–2nd quarter	0.123	0.634	33			0.194	0.997		
1st–3rd quarter	−0.231	0.634	33			−0.365	0.983		
1st–4th quarter	−1.136	0.634	33			−1.791	0.296		
2nd–3rd quarter	−0.354	0.634	33			−0.558	0.944		
2nd–4th quarter	−1.259	0.634	33			−1.984	0.214		
3rd–4th quarter	−0.904	0.634	33			−1.426	0.493		

There was also no significant interaction effect between time and body mass. Estimated marginal trends with a Tukey adjustment confirmed this; there were no significant differences in the allometric scaling of locomotory energetics between any of the four time periods. *P*-value adjustment, Tukey method for comparing a family of four estimates.

**Table 5.** One-way analysis of covariance indicates that while both time and body mass are significant determinants of locomotory energetics in *H. glaber*, there is no significant interaction effect

	Estimate	s.e.	d.f.	SS	MS	*t-*ratio	*P*	*F*	Pr(>*F*)
Body mass			1	17,206	17,206			6.449	0.034*
Time			3	2654	884.8			6.158	0.003*
Time×Body mass			3	1183	394.3			2.744	0.065
Contrast									
1st–2nd quarter	0.335	0.714	24			0.47	0.965		
1st–3rd quarter	1.041	0.714	24			1.459	0.477		
1st–4th quarter	1.887	0.714	24			2.645	0.063		
2nd–3rd quarter	0.706	0.714	24			0.989	0.757		
2nd–4th quarter	1.552	0.714	24			2.176	0.158		
3rd–4th quarter	0.846	0.714	24			1.186	0.641		

Estimated marginal trends with a Tukey adjustment confirmed this; there were no significant differences in the allometric scaling of locomotory energetics between any of the four time periods. *P*-value adjustment, Tukey method for comparing a family of four estimates.

## DISCUSSION

We found that *F. damarensis* exhibits a 1-fold (ca. 100%) and *H. glaber* a 2-fold (ca. 216%), increase in their respective RMR in response to sustained submaximal locomotion (10 cm s^−1^). While *F. damarensis* exhibits a comparable allometric scaling of metabolic rate both at rest and during locomotion, there is a 3-fold increase in the allometric scaling exponent of *H. glaber* during locomotion.

Subterranean environments represent an inherent challenge for species to not only inhabit but also navigate and move through; an absence of light, intermittent resources and increased energetic costs of digging through resistive substrates all characterise such environments (Nevo, 1979; Burda et al., 2007; Jonz et al., 2016). The challenge of the underground environment is further compounded by reduced gas ventilation, which can prompt hypoxic and hypercapnic conditions Nevo, 1979). In response, subterranean species typically exhibit an evolutionarily convergent suite of behavioural, morphological and physiological adaptations, which enable the successful exploitation of this ecological niche (Nevo, 1979). One such response typical of subterranean rodents is a lower RMR than terrestrial rodents of comparable size, and in specific clades, such as African mole-rats, lower body temperature and higher thermal conductance (McNab, 1979; Buffenstein, 2000; Nevo, 2007), which is believed to help mitigate heat stress generated in a closed burrow system (the thermal stress hypothesis; McNab, 1966). These species may also exhibit a reduced RMR on account of hypoxic and hypercapnic conditions (the respiratory stress hypothesis; Arieli, 1979) or to compensate for the high energetics costs of subterranean foraging (the cost of burrowing hypothesis; Vleck, 1979; Lovegrove, 1987; Lovegrove and Wissel, 1988; Luna and Antinuchi, 2007). Furthermore, the risk-sensitive metabolism hypothesis posits that increased sociality amongst African mole-rat species coupled with a low RMR would be favoured when the energetics associated with foraging for irregularly distributed resources is high (Lovegrove and Wissel, 1988; Merchant et al., 2024c). Indeed, *F. damarensis* and *H. glaber* exhibit mass-specific RMR that, respectively, is 43% and 57% lower than that predicted for rodents (Lovegrove, 1986). Although burrowing is energetically costly, ultimately, the benefits – access to subsurface food resources, food caching, protection from bioclimatic extremes and predation, nesting and hibernation, as well as communication facilitated by enhanced acoustics (Bennet-Clark, 1987; White et al., 2006b; Burda et al., 2007; Zelová et al., 2007; Horner and Biknevicius, 2010) – must outweigh the associated costs.

### Locomotory energetics of submaximal locomotion over and above RMR

While the energetic cost of digging in *F. damarensis* and *H. glaber* has previously been identified to be between three and five times greater than their respective RMR, this study reveals that sustained submaximal locomotion prompts a 1-fold increase in energetic expenditure in *F. damarensis*, whereas *H. glaber* exhibits a 2-fold increase. What is conclusive is that, somewhat unsurprisingly, for both species, the energetic demand associated with burrowing is greater than that of locomotion. One potential limitation of the present study was that locomotory energetics were measured under normoxic conditions. However, new tunnels are typically excavated by a single individual at relatively shallow depths, which likely results in normoxic conditions (Roper et al., 2001), similar to the environment under which the animals were measured in the present study. Roper et al. (2001) found minimum and mean oxygen concentrations of 19.9–20.4%, for example, from *F. damarensis* burrows.

While a strong scaling relationship exists between the locomotory costs of movement and body mass, i.e. species with larger body masses tend to have greater absolute energetic requirements to move a given distance (Halsey and White, 2012); per unit body mass, larger species tend to have a reduced COT (Heglund et al., 1982; Full, 1989). Indeed, *H. glaber* had a higher COT than *F. damarensis* (Table 1). Based on Alexander (2005), the COT for *F. damarensis* and *H. glaber* is predicted to be approximately 35 and 40 J m^−1^ kg^−1^, respectively, compared with the 129 and 161 J m^−1^ kg^−1^ measured in the present study. These measurements refute our initial proposition that mole-rats should have lower costs of transport because of the low stability requirements from burrow living. The high COT recorded is likely due to the unsteady nature of mole-rat gait, and their movement not being optimised for economy. Moreover, the highest costs are likely to be because the limbs of mole-rats are adapted for different activities, and not optimised for economy of body weight support during locomotion. Horner et al. (2016) found, for example, that net COT increased by 30% and 17% for domestic ferrets (*Mustela putorius furo*) and degus (*Octodon degu*), respectively, when having to crouch to move through tunnels, as opposed to moving unconstrained.

### Allometric scaling in response to sustained locomotion

Our study found no significant differences in the respective allometric scaling exponents associated with locomotory energetics of either species across the four time periods. Scaling exponents ranged between 0.9 and 2.1 for *F. damarensis* and between 1.7 and 3.6 for *H. glaber*. Despite this, appreciable differences arose when compared the allometric scaling exponent of these species at rest (i.e. during RMR assessments; Fig. S1): 1.47 for *F. damarensis* and 0.57 for *H. glaber*. While *F. damarensis* exhibits a comparable scaling exponent during rest and sustained locomotion, *H. glaber* exhibits more than a 3-fold increase in allometric scaling exponent during locomotion compared with that of resting animals. The large increase in allometric scaling indicates that *H. glaber* may respond differently to locomotion; specifically, that heavier animals respond differently to sustained locomotion. That heavier individuals show a more pronounced response suggests that there is a greater increase in metabolic rate with increasing body mass (i.e. as animals get heavier their metabolic rate increases more). However, it is worth noting that, again, these interspecies differences may be driven by the considerable size and mass differences between these two species; had *H. glaber* been assessed at a lower speed, proportional to their smaller body conformation, the allometric scaling of locomotory energetics may have been more comparable. Alternatively, the drop in RMR over time may be required to avoid overheating or exercise-induced hyperthermia (Hart et al., 2021; Jacobs et al., 2022a,b). Furthermore, the absence of allometric scaling in *F. damarensis* may be attributed to their need to avoid exercise-induced hyperthermia. Larger individuals tend to generate and retain heat at a higher rate compared with smaller counterparts. In the case of *F. damarensis*, their possession of hair limits their ability to efficiently dissipate heat, unlike the hairless *H. glaber*. Consequently, larger *F. damarensis* individuals must regulate their metabolic rate to mitigate the risk of exercise-induced hyperthermia (Hart et al., 2021; Jacobs et al., 2022a,b; Grenfell et al., 2024). The metabolic adjustment likely results in the absence of allometric scaling in *F. damarensis*, contrasting with *H. glaber*, which does not face the same thermal regulation challenges due to their lack of hair.

### Limitations

As has been previously noted, under laboratory conditions, the determined energetic costs of burrowing and locomotion are poor approximations of conditions contended by conspecifics in a wild, heterogeneous environment (Du Toit et al., 1985; Shepard et al., 2013). This is rarely a reflection of poor experimental design, it simply represents an unavoidable constraint for assessments within a captive environment. For example, there are challenges replicating the soil compactness and humidity that wild animals contend with whilst burrowing, which also vary seasonally in response to changing climatic conditions (e.g. increased precipitation). Furthermore, respirometry assessments of burrowing require the substrate to be dry to enable the measurement of gas exchange. However, in the wild, environments that experience high seasonal bioclimatic changes can result in water-logged or sun-baked substrates, forcing animals to move through and excavate either a heavy wet or hard compacted substrate. Burrowing in such conditions represents an even greater energetic cost. Indeed, for *Ctenomys talarum*, the energetic cost of digging through hard soil is 52.6% greater than that for digging through soft soil; for subterranean rodents, substrate hardness appears to be an important governing factor affecting burrowing efficiency (Luna and Antinuchi, 2006). Indeed, this may be one reason that African mole-rats are known to extend tunnel systems during the rainy seasons when the substrate is softer (Herbst and Bennett, 2006; Van Daele et al., 2009). Furthermore, most energetic assessments of locomotion utilise a treadmill, which, with its uniformly firm and flat surface, inadequately simulates the conditions that wild animals experience underfoot. Indeed, locomotory energetics increase when animals move across substrates that have a proclivity to move, such as sand and snow (Karasov, 1992).

The speed of the treadmill (10 cm s^−1^) was decided upon based on the apparent preference for sustained movement at this speed; lower speeds resulted in a discontinuous and erratic movement, while higher speeds resulted in refusals to move. Such ‘preferred’ speeds have been used multiple times in prior studies, for the determination of exercise metabolic expenditure (Heglund et al., 1982). While these species are morphologically dissimilar in both their mean body mass (124.0±32.8 g *F. damarensis* and 31.3±9.7 g *H. glaber*) and body length, this speed appeared to suit both species. Despite the smaller morphological conformation of *H. glaber*, anecdotal observations indicate that this species is adept and agile, and has a predisposition for moving quickly. A period of habituation may have enabled the locomotory assessment of animals at incrementally increasing speeds.

## Supplementary Material

10.1242/jexbio.249875_sup1 Supplementary information

Table S1. Full Data set for “The locomotory energetics of sustained submaximal locomotion in two African mole-rat species *(Fukomys damarensis and Heterocephalus glaber)*”
