# Supplementary material for: Metabolic expenditure of submaximal locomotion in naked mole-rats (Heterocephalus glaber) and Damaraland mole-rats (Fukomys damarensis)
Source: J Exp Biol. 2025 Jun 25;228(12):jeb249875. doi: 10.1242/jeb.249875 (PMC12268170; doi:10.1242/jeb.249875)
Supplement: Supplementary information [file jexbio-228-249875-s1.pdf]

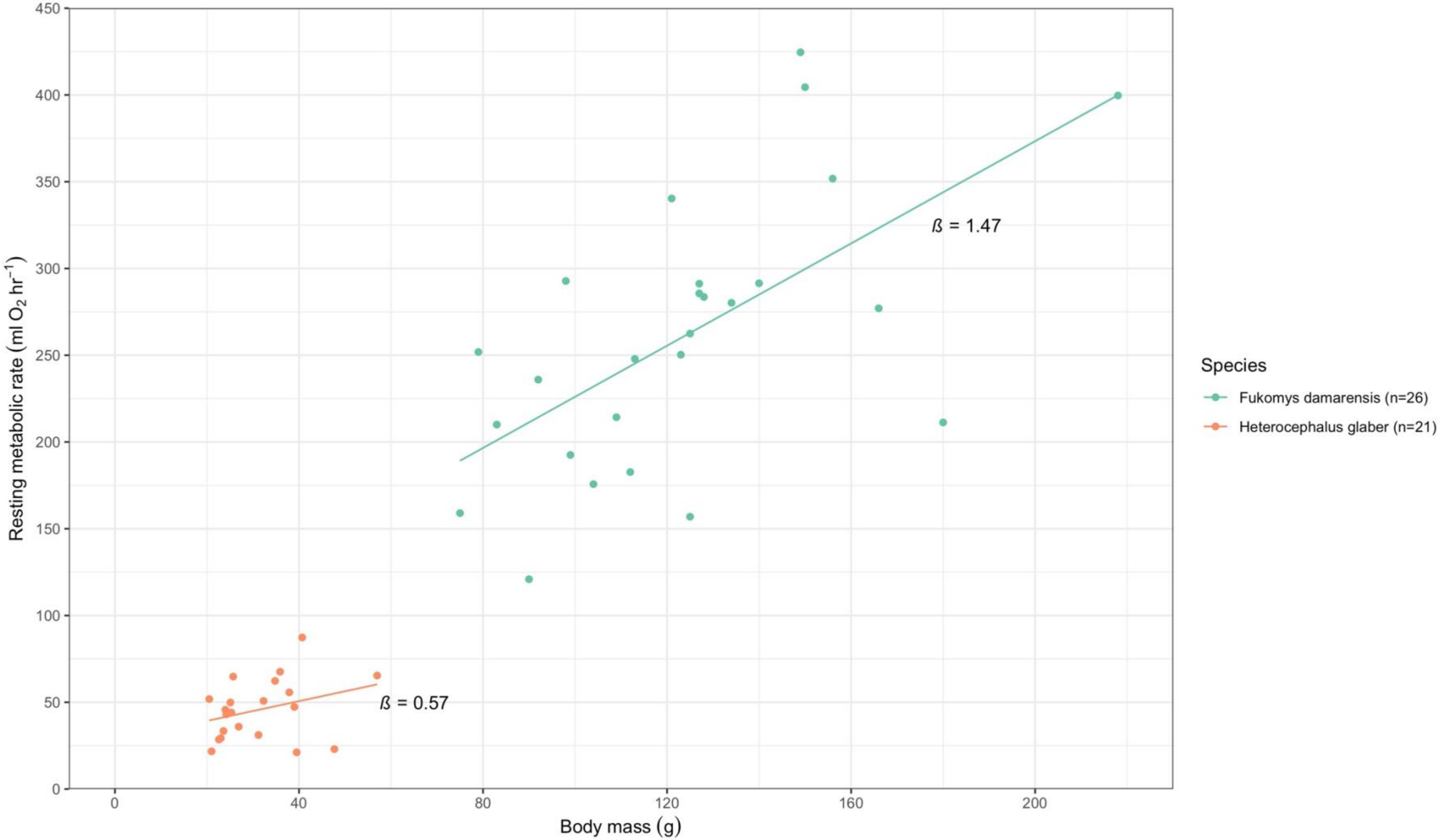

Fig. S1. The allometric scaling of resting metabolic rate (ml O_2 hr^{-1}) in two species of African mole-rats (*Fukomys damarensis* and *Heterocephalus glaber*). The scaling exponent (*ß*) for each species is indicated.

Table S1. Full Data set for "The locomotory energetics of sustained submaximal locomotion in two African mole-rat species (*Fukomys damarensis* and *Heterocephalus glaber)"*

Available for download at
https://journals.biologists.com/jeb/article-lookup/doi/10.1242/jeb.249875#supplementary-data
